# The Efficacy and Safety of Subcutaneous Ixekizumab for the Treatment of Axial Spondylarthritis: A Systematic Review and Meta-Analysis

**DOI:** 10.7759/cureus.35360

**Published:** 2023-02-23

**Authors:** Ziyad Alzahrani, Bader A Bashrahil, Rakan Alzahrani, Fayez Alharthy

**Affiliations:** 1 College of Medicine, King Saud Bin Abdulaziz University for Health Sciences College of Medicine, Jeddah, SAU; 2 Internal Medicine/Rheumatology, King Saud Bin Abdulaziz University for Health Sciences College of Medicine, Jeddah, SAU; 3 Medicine, King Abdullah International Medical Research Center, Jeddah, SAU; 4 Medicine, King Abdulaziz Medical City, Jeddah, SAU

**Keywords:** rct, spondylarthritis, ankylosing spondylarthritis, randomized controlled trial (rct), ixekizumab

## Abstract

Axial spondylarthritis (axSpA) is a progressive inflammatory condition that is treated with various management options. Interleukin-17A (IL-17A) inhibitors are a novel therapeutic option that demonstrates both efficacy and safety. This systematic review and meta-analysis evaluated the effectiveness of ixekizumab and its safety compared to a placebo.

Medline, ScienceDirect, EBSCO, and Cochrane Central Register of Controlled Trials (CENTRAL) were searched. We included randomized control trials (RCTs) that assessed the efficacy and safety of ixekizumab versus the placebo. The GRADE (Grading of Recommendations, Assessment, Development, and Evaluations) assessment was utilized to evaluate the certainty of evidence. The revised Cochrane risk of bias tool for randomized trials was used to assess the risk of bias.

Four RCTs (n=1016) met the eligibility criteria. All included studies had a low risk of bias. Significant improvements in the Assessment of Spondylarthritis International Society response for 40% improvement (ASAS40) (RR = 2.39, 95% CI 1.72-3.31, P < 0.01, I2 = 23%), Ankylosing Spondylitis Disease Activity Score (ASDAS) (SMD= -9.28 95% CI -12.31- (-6.25), P < 0.01, I2=97%), and Spondylarthritis Research Consortium of Canada (SPRACC score) (SMD= -5.82 95% CI -7.16- (-4.47), P < 0.01, I2=94%) were noted in comparison to placebo. Regarding safety, there was an insignificant increase in risk for serious adverse events (SAEs) (RR = 1.19, 95% CI 0.45-3.14, P = 0.73, I2 = 0%). Additionally, significant nonserious adverse events (NSAEs) (RR = 1.54, 95% CI 1.19-1.99, P = 0.001, I2 = 0%) were noted for the ixekizumab arm. No mortality events were detected in both arms.

Ixekizumab, which demonstrates significant improvement in all efficacy endpoints, is a promising management option for axSpA patients who fail non-steroidal anti-inflammatory drugs (NSAIDs) therapy. However, the significant risk of developing adverse events hinders its utilization. More high-quality RCTs with larger sample sizes and prolonged follow-up periods are warranted to further assess this treatment option.

## Introduction and background

Axial spondylarthritis is a group of disorders that includes both radiographic axSpA and non-radiographic axSpa [1,2]. Ankylosing spondylitis, also known as radiographic axSpA, is a type of spondyloarthropathy that is characterized by its inflammatory involvement of the spine, sacroiliac joint, and nearby tissues [1]. While similar in signs and symptoms, the presence of radiographical changes is the main differentiating point. As the disease progresses, it will cause further inflammation in the affected joints, predisposing the joints to be more prone to ankylosis and calcification [1]. It is a disease with multifactorial etiology, including genetics, and immunological aspects. The worldwide prevalence of AS was estimated to be 0.5% and is more commonly seen in men [2]. Moreover, the prevalence is increased in the human leukocyte antigen (HLA)-B27-positive population, as 90% of AS patients were found to have positive HLA-B27 testing [1,2]. The lack of efficacious disease-modifying agents and the ambiguous nature of the disease hinders the treatment options for axSpA [1].

Biological disease-modifying anti-rheumatic drugs (bDMARDs) remain the only treatment options for axSpA patients who do not respond to nonsteroidal anti-inflammatory medications [1]. The Janus kinase 1 inhibitor is another management option that appears to be an efficacious treatment [3]. Due to the slow nature of the disease progression and the absence of definitive diagnostic tests, trials for axSpA treatment are hindered by an indefinite axSpA diagnosis [1]. Both the disease activity and radiographic measurements provide moderate prognostic and diagnostic values [4,5]. The Ankylosing Spondylitis Disease Activity Score (ASDAS) has been shown to demonstrate the inflammatory status of axSpA better than the Bath Ankylosing Spondylitis Disease Activity Index (BASDI), and both are used to assess disease severity [1,4]. Moreover, the Assessment of Spondylarthritis International Society response for 40% improvement was used to estimate the clinical response. Radiographic measurements, such as the Spondylarthritis Research Consortium of Canada MRI score, offer an objective evaluation of the inflammation of the sacroiliac joint [5].

Non-steroidal anti-inflammatory drugs (NSAIDs) are the first-line treatment for the majority of axSpA patients, but this treatment has been shown to have some limitations. The continuous use of NSAIDs is correlated with an increased risk of gastrointestinal, renal, and cardiovascular major side effects [6]. Ixekizumab is one of the most novel interleukin-17A (IL-17) inhibitors and has been used recently for the treatment of axSpA [7]. IL-17A is an important cytokine that plays a role in the pathogenesis of axSpA [7,8]. It is theorized that the blockage of IL-17A is useful in the treatment of axSpA [8]. IL-17A has been discussed in several articles for the management of AS, including randomized controlled trials (RCTs) [8-11].

This systematic review and meta-analysis were performed to evaluate the safety and efficacy of subcutaneous ixekizumab compared to placebo. Safety was assessed by measuring the incidence of serious adverse events (SAEs), nonserious adverse events (NSAEs), and mortality. For efficacy, patients were assessed for the ASAS40, ASDAS, and SPARCC scores.

## Review

Materials and methods

This study was registered prior to a preliminary search in alignment with PROSPERO (CRD42022352274) and utilized the Preferred Reporting Items for Systematic Reviews and Meta-Analysis (PRISMA) checklist. We declare that all data are available in this article. Furthermore, this study did not require ethical approval, as the data in this study were already published previously.

Eligibility criteria

This review only included RCTs that compared ixekizumab to placebo and evaluated its effectiveness through ASAS40, ASDAS, and SPARCC. Patients who were diagnosed with active axSpA or who fulfilled modified New York criteria were included [12]. TNFi-naïve patients and patients who did not achieve sufficient clinical improvement or were intolerant to TNFi treatment (TNFi-IR) were also included. However, RCTs that included confounding conditions affecting the sacroiliac joints were excluded. In our study, a meta-analysis was conducted assessing the ASAS40, ASDAS, and SPARCC score regarding clinical improvement, which was the primary efficacy endpoints. The incidence of SAEs, NSAEs, and mortality was measured by their frequency during the trials. The frequency of ixekizumab administration, in some RCTs, was prescribed in different time intervals (i.e., 80 mg Q2W and ixekizumab 80 mg Q4W), which were included in the subgroup analysis.

Search strategy

Our study systematically searched the Medline, ScienceDirect, EBSCO, and Cochrane Central Register of Controlled Trials (CENTRAL) databases from database initiation to August 8, 2022, without any restriction on date or language. On August 8, 2022, we searched all included databases with keywords including “Ankylosing spondylitis”, “Axial spondylarthritis”, “LY2439821”, and “Ixekizumab”. Manual searches of reference lists from recent systematic reviews and published studies were also included. The references of the included RCTs were checked for any relevant RCTs that were missed during the systematic search process. The search strategy that was utilized is provided in Table 1.

**Table 1:** Search strategy

Medline
The database was searched on: August 8, 2022, n=92.
((((ankylosing spondylitis or axial spondyloarthritis)) AND (((((((anti‐interleukin‐17) OR anti‐IL‐17) OR IL17 receptor blockade) OR anti‐IL17R) OR ixekizumab)
Cochrane Library
The database was searched on August 8, 2022, n=208.
1- (Ankylosing spondylitis or Bechterew Disease or Marie Struempell Disease or axial spondyloarthritis).af.
2-LY2439821 or Taltz or Ixekizumab or anti interleukin 17).af.
3- (Randomized Controlled Trial or Clinical Trial or RCT or Trial).af.
4- 1 and 2 and 3
EBSCO
The database was searched on August 8, 2022, n=12.
1- (Ankylosing spondylitis or Bechterew Disease or Marie Struempell Disease or axial spondyloarthritis).af.
2-(LY2439821 or Taltz or Ixekizumab or anti interleukin 17).af
3- (Randomized Controlled Trial or Clinical Trial or RCT or Trial).af.
4- S1 and S2 and S3
ScienceDirect
The database was searched on August 8, 2022, n=136.
(Ankylosing spondylitis or axial spondyloarthritis AND Ixekizumab OR LY2439821 OR Taltz OR anti interleukin 17 AND Randomized Controlled Trial)

Study selection and data extraction

Independently, two reviewers (ZA and BB) in duplicate performed title and abstract screening, full-text assessment, and data extraction for all the RCTs with the eligibility criteria. Disagreements were discussed with a third reviewer or resolved through consensus prior to further advancement in the process.

Meta-analysis

Data analysis was performed using RevMan (Review Manager) version 5.3 (Cochrane Collaboration). All statistical analyses were performed using the random-effects model. A 95% confidence level and P < 0.05 as a borderline were set for statistical significance. The statistical heterogeneity was assessed using the I2 and P values of the chi-square test. The assessment of dichotomous outcomes (ASAS40, SAEs, NSAEs, and mortality) was represented as risk ratios (RRs) and pooled using the inverse variance weighting method. For continuous outcomes, the mean change in the ASDAS and SPARCC scores was utilized, and their efficacy was measured through the standardized mean difference (SMD). Moreover, sensitivity analysis was undertaken to assess the robustness of the results. The frequency of ixekizumab administration was included in the subgroup analysis in comparison to the placebo group. The Grading of Recommendations Assessment, Development, and Evaluation (GRADE) criteria were used to assess the quality of evidence of all reported outcomes.

Risk of bias assessment

Two reviewers (ZA and RA), independently and together, used the Revised Cochrane risk of bias tool to assess the risk of bias in the eligible RCTs. All studies included were reviewed and scored either as high risk, low risk, or some concerns. Differences between the reviewers were resolved through discussion until an agreement was achieved.

Results

**Figure 1:**
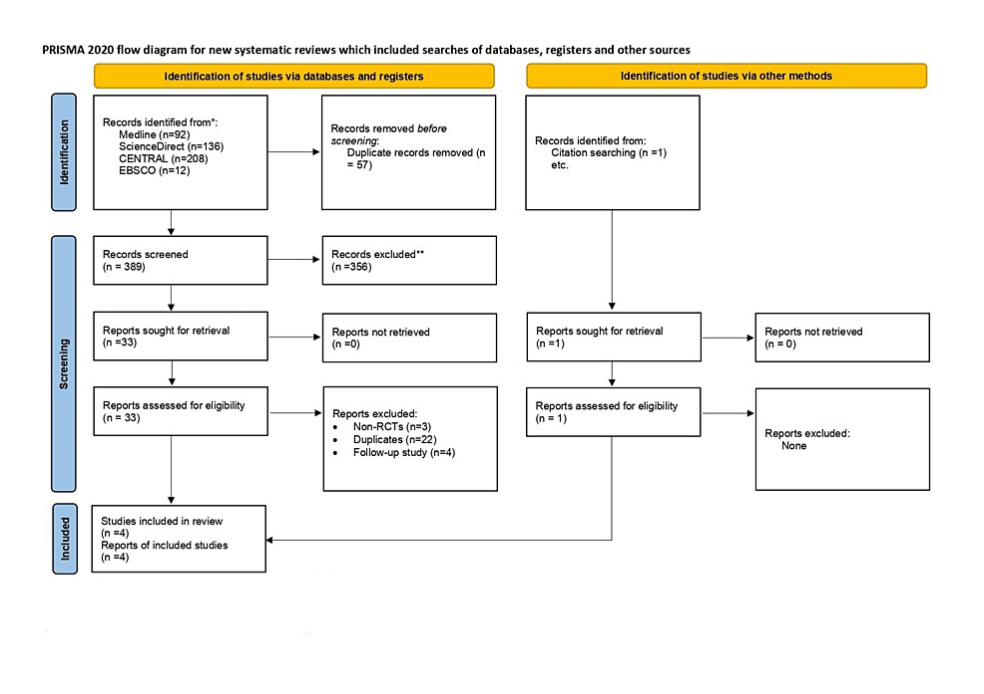
PRISMA flow chart of the included studies CENTRAL: Cochrane Central Register of Controlled Trials, RCT: randomized controlled trial, PRISMA: Preferred Reporting Items for Systematic Reviews and Meta-Analysis Page MJ, McKenzie JE, Bossuyt PM, Boutron I, Hoffmann TC, Mulrow CD, et al. The PRISMA 2020 statement: an updated guideline for reporting systematic reviews. BMJ 2021;372:n71. doi: 10.1136/bmj.n71. For more information, visit: http://www.prisma-statement.org/

A flowchart of all the studies included with a detailed inclusion process and justification for excluded studies is demonstrated in Figure 1. Upon initial search, 445 studies were included. Duplicates were identified as 57 studies, resulting in the screening of 388 studies. Out of 388 studies, 33 studies were included in the full article screening, as all excluded studies did not match the eligibility criteria. During full article screening, 29 articles were excluded due to being duplicates, being follow-up studies, or being non-RCTs. Ultimately, four studies were included in the meta-analysis. All four studies had matching arms of ixekizumab versus placebo.

Trial characteristics

**Figure 2:**
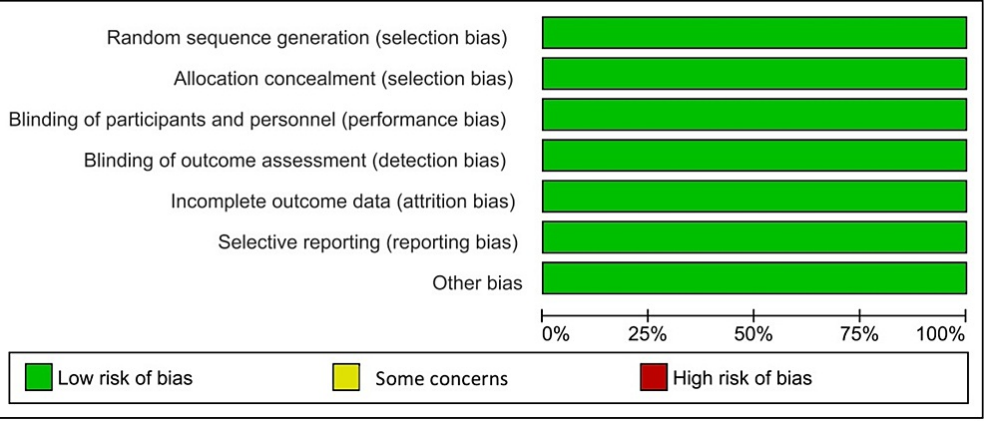
Risk of bias assessment graph

The trials in this study included 1016 patients. The mean age for these studies ranged from 33.9 to 46.1. Regarding sex, male patients comprised 728 participants (71.6%) of all 1016 participants. The majority were classified as either not Hispanic or Latino, constituting 71%, and the remainder were either Hispanic or Latino or were classified as unknown. Trials characteristics can be seen in Table 2. All included RCTs had a low risk of bias (Figure 2).

**Table 2:** Characteristics of included trials RCT: randomized controlled trial, IXE80QW2: ixekizumab 80 mg once every two weeks, IXE80Q4W: ixekizumab 80 mg once every four weeks, r-xSpA: radiographic axial spondyloarthritis, nr-axSpA: non-radiographic axial spondyloarthritis

Study name	Study design	Mean follow-up	Study groups	Number of patients	Demographic data (i.e. Gender, Age)	Exposure assessment (compliance)	Duration of exposure	Study population
NCT02757352 (COAST-X) [8]	RCT	52 weeks	IXE80Q2W (80 mg), IXE80Q4W (80 mg), Placebo	IXE80Q2W (n=102), IXE80Q4W (n=96), Placebo (n=105)	Gender (Male; n= 143, Female; n= 160) Age (IXE80Q2W 40.0 ± (12.1), (IXE80Q4W 40.9 ± (14.47), (Placebo 39.9 ± (12.36)	Study Drug Administration Log at each visit	52 weeks	nr-axSpA,
NCT02696798 (COAST-W) [9]	RCT	52 weeks	IXE80Q2W (80 mg), IXE80Q4W (80 mg), Placebo	IXE80Q2W (n=98), IXE80Q4W (n=114), Placebo (n=104)	Gender (Male; n= 253, Female; n= 63) Age (IXE80Q2W 44.2 ± (10.79), (IXE80Q4W 47.4 ± (13.36), (Placebo 46.6 ± 12.72))	Study Drug Administration Log at each visit	52 weeks	r-xSpA
NCT02696785 (COAST-V) [10]	RCT	52 weeks	IXE80Q2W (80 mg), IXE80Q4W (80 mg), Adalimumab (40 mg), Placebo	IXE80Q2W (n=83), IXE80Q4W (n=81), Adalimumab (n=90), Placebo (n=87)	Gender (Male; n= 276, Female; n= 64) Age (IXE80Q2W 41.3 ± (11.17), (IXE80Q4W 41.0 ± (12.13), (Adalimumab 41.8 ± (11.44), (Placebo 42.7 ± (12.01)	Study Drug Administration Log at each visit	52 weeks	r-xSpA
NCT04285229 [11]	RCT	16 weeks	IXE80Q4W (80 mg), Placebo	IXE80Q4W (n=74), Placebo (n=73)	Gender (Male; n= 129, Female; n= 18) Age (IXE80Q4W 33.5 ± (8.89), (Placebo 34.4 ± (8.98)	Study Drug Administration Log at each visit	16 weeks	r-xSpA

Assessment of Spondylarthritis International Society 40 response

**Figure 3:**
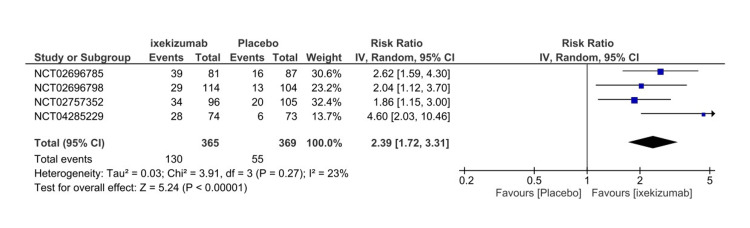
Assessment of Spondylarthritis International Society 40 response forest plot CI, confidence interval; IV, inverse variance; RR, risk ratio. [8-11]

**Figure 4:**
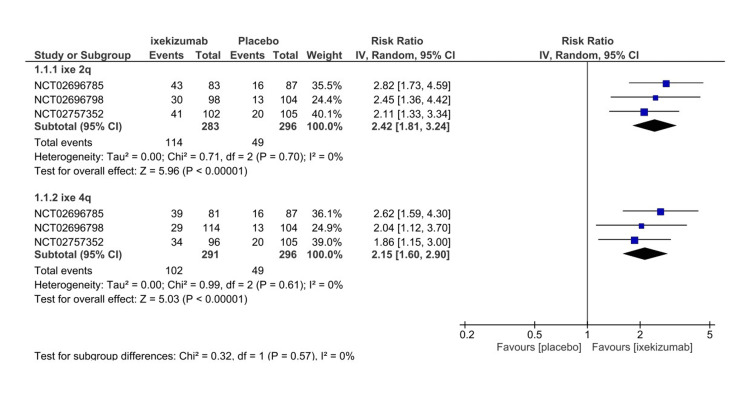
Assessment of Spondylarthritis International Society 40 response subgroup forest plot CI, confidence interval; IV, inverse variance; RR, risk ratio; Q2, every 2 weeks; Q4, every 4 weeks. [8-10]

Four RCTs that measured ASAS40 at different timeframes (e.g., 16 weeks, 52 weeks) are Included in our meta-analysis to assess the efficacy. The measurement of ASAS40 included only patients who completed a trial of 16 weeks. The ixekizumab Q4W arm demonstrated a significantly higher occurrence rate than the placebo arm for ASAS40 (RR = 2.39, 95% CI 1.72-3.31, P < 0.01, I2 = 23%) (Figure 3). For the subgroup analysis, we included three RCTs that compared the ixekizumab administration at different time intervals. Significant events were observed in both arms of IXE80Q2W and IXE80Q4W versus placebo (RR = 2.42, 95% CI 1.81-3.24, P < 0.01, I2 = 0%) and (RR = 2.15, 95% CI 1.60-2.90, P < 0.01, I2 = 0%), respectively (Figure 4) The GRADE assessment was considered high for efficacy (Table 3).

**Table 3:** GRADE criteria assessment №: population size; CI: confidence interval; RR: risk ratio; SMD: standardised mean difference; ASAS40: Assessment of Spondylarthritis International Society 40 response; ASDAS: Ankylosing Spondylitis Disease Activity Score; SPARCC: Spondyloarthritis Research Consortium of Canada score; GRADE: Grading of Recommendations Assessment, Development and Evaluation

Certainty assessment							№ of patients		Effect		Certainty	Importance
№ of studies	Study design	Risk of bias	Inconsistency	Indirectness	Imprecision	Other considerations	Ixekizumab	placebo for ankylosing spondylitis	Relative (95% CI)	Absolute (95% CI)		
Number of Participants Achieving an (ASAS40) Response in 16 weeks												
4	randomized trials	not serious	not serious	not serious	not serious	strong association	130/365 (35.6%)	55/369 (14.9%)	RR 2.39 (1.72 to 3.31)	207 more per 1,000 (from 107 more to 344 more)	⨁⨁⨁⨁ High	IMPORTANT
Change From Baseline in ASDAS in 16 weeks												
4	randomized trials	not serious	serious^a,b^	not serious	not serious	none	365	369	-	SMD 9.28 lower (12.31 lower to 6.25 lower)	⨁⨁⨁◯ Moderate	NOT IMPORTANT
Change From Baseline in Magnetic Resonance Imaging (MRI) of (SPARCC) Score (follow-up: mean 16 weeks)												
4	randomized trials	not serious	serious	not serious	not serious	none	365	369	-	SMD 5.82 lower (7.16 lower to 4.47 lower)	⨁⨁⨁◯ Moderate	NOT IMPORTANT
Serious Adverse Events												
4	randomized trials	not serious	not serious	not serious	not serious	none	9/365 (2.5%)	7/367 (1.9%)	RR 1.19 (0.45 to 3.14)	4 more per 1,000 (from 10 fewer to 41 more)	⨁⨁⨁⨁ High	IMPORTANT
Non-serious Adverse Events												
4	randomized trials	not serious	not serious	not serious	not serious	none	107/365 (29.3%)	71/367 (19.3%)	RR 1.54 (1.19 to 1.99)	104 more per 1,000 (from 37 more to 192 more)	⨁⨁⨁⨁ High	IMPORTANT
Deaths												
4	randomized trials	not serious	not serious	not serious	not serious	none	0/365 (0.0%)	0/367 (0.0%)	not pooled		⨁⨁⨁⨁ High	NOT IMPORTANT

Ankylosing Spondylitis Disease Activity Score

**Figure 5:**
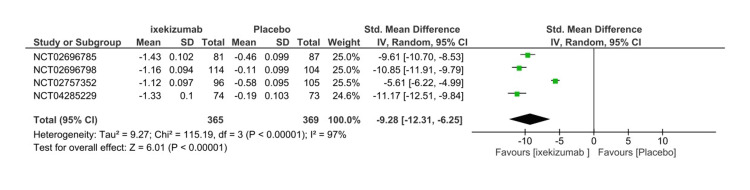
Ankylosing Spondylitis Disease Activity Score forest plot CI, confidence interval; IV, inverse variance; SMD, standardized mean difference; SD, standard deviation [8-11]

**Figure 6:**
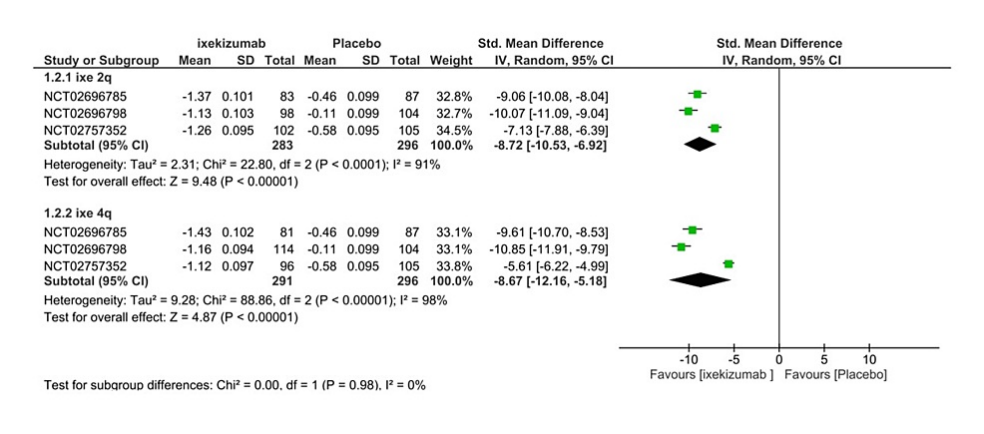
Ankylosing Spondylitis Disease Activity Score subgroup forest plot IV, inverse variance; SMD, standardized mean difference; SD, standard deviation; Q2, every 2 weeks; Q4, every 4 weeks [8-10]

All RCTs included the assessment of the mean change in ASDAS at 16 weeks, which was used to estimate the efficacy. The ASDAS comprises five different domains: C-reactive protein (CRP), total back pain, duration of morning stiffness, patient global, and peripheral pain. Higher scores indicate an increase in disease activity. A significant decrease in ASDAS score was found in the IXE80Q4W arm versus placebo (SMD= -9.28 95% CI -12.31- (-6.25), P < 0.01, I2=97%) (Figure 5). To explain the heterogeneity, a subgroup analysis was performed including three RCTs. Both IXE80Q2W (SMD= -8.72 95% CI -10.53- (-6.92), P < 0.01, I2=91%) and IXE80Q4W (SMD= -8.67 95% CI -12.16- (-5.18), P < 0.01, I2=97%) significantly decreased ASDAS in comparison to placebo (Figure 6) The GRADE assessment was considered moderate for efficacy (Table 3).

Spondyloarthritis Research Consortium of Canada score

**Figure 7:**
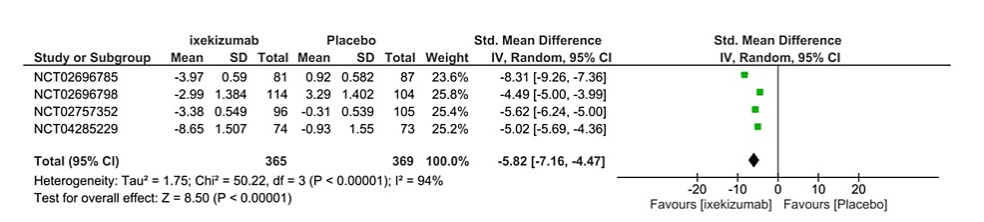
Spondyloarthritis Research Consortium of Canada scores forest plot CI, confidence interval; IV, inverse variance; SMD, standardized mean difference; SD, standard deviation. [8-11]

**Figure 8:**
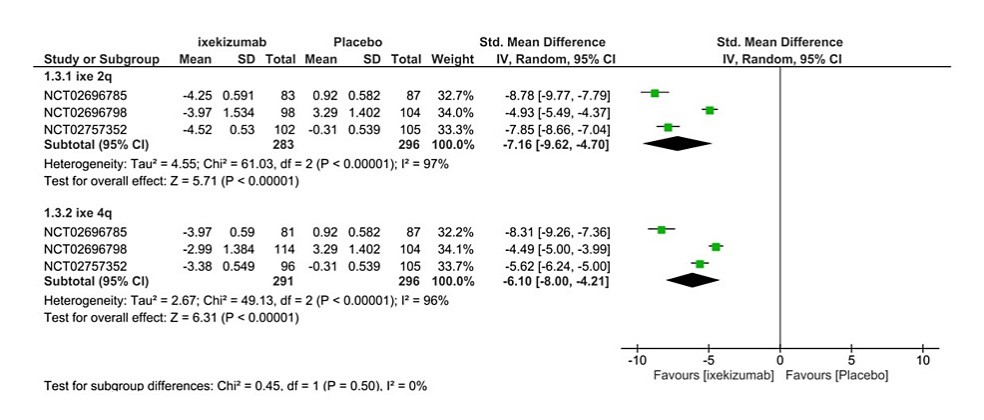
Spondyloarthritis Research Consortium of Canada scores subgroup forest plot CI, confidence interval; IV, inverse variance; SMD, standardized mean difference; SD, standard deviation; Q2W, every 2 weeks; Q4W, every 4 weeks [8-10]

The SPARCC score is one of the primary efficacy endpoints utilized to assess efficacy at 16 weeks. The SPARCC score can range from 0 to 72, in which a higher score indicates the progression of the disease. Bone marrow edema is scored for the left sacroiliac joint (SIJ) and right SIJ, in which each side has six slices. Moreover, each slice contains six scoring units that can be scored from zero to one. Significant improvement in the SPARCC score was noted in the ixekizumab Q4W arm (SMD= -5.82 95% CI -7.16- (-4.47), P < 0.01, I2=94%) (Figure 7). Further subgroup analysis was performed to explain the high heterogeneity. Both IXE80Q2W (SMD= -7.16 95% CI -10.53- (-9.62), P < 0.01, I2=917%) and IXE80Q4W (SMD= -6.10 95% CI -8.00- (-4.21), P < 0.01, I2=96%) significantly decreased the SPARCC score in comparison to placebo (Figure 8). The GRADE assessment was considered moderate for efficacy (Table 3).

Serious adverse events

**Figure 9:**
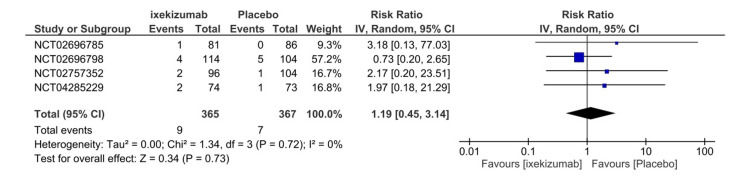
Serious adverse events forest plot CI, confidence interval; IV, inverse variance; RR, risk ratio [8-11]

**Figure 10:**
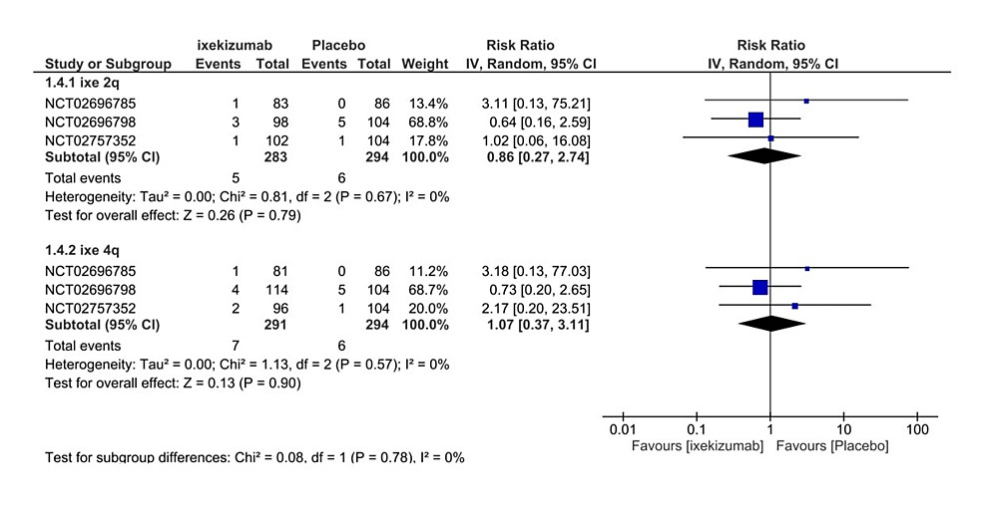
Serious adverse events subgroup forest plot CI, confidence interval; IV, inverse variance; RR, risk ratio; Q2, every 2 weeks; Q4, every 4 weeks [8-10]

Four RCTs included in the quantitative analysis assessed the frequency of SAEs following the first administration until the participants’ last follow-up. SAEs were defined as any event according to the study evaluator that contributed to death, a life-threatening event, inpatient hospitalization, prolongation of existing hospitalization, the results in an ongoing or significant incapacity, or a congenital anomaly or birth defect in an offspring of a study participant. The total number of events for both the ixekizumab Q4W arm and the placebo arm was 17 events, of which nine were attributed to ixekizumab. Furthermore, the placebo arm demonstrated a lower risk of SAEs, which was insignificant (RR = 1.19, 95% CI 0.45-3.14, P = 0.73, I2 = 0%) (Figure 9). In addition, subgroup analysis of ixekizumab Q4W (IXE80Q4W) and ixekizumab Q2W (IXE80Q2W) versus placebo indicated that ixekizumab Q2W had a lower tendency to induce SAEs (RR = 0.86, 95% CI 0.27-2.75, P = 0.80, I2 = 0%) (Figure 10). SAEs’ GRADE evaluation leads to high certainty of evidence (Table 3).

Nonserious adverse events

**Figure 11:**
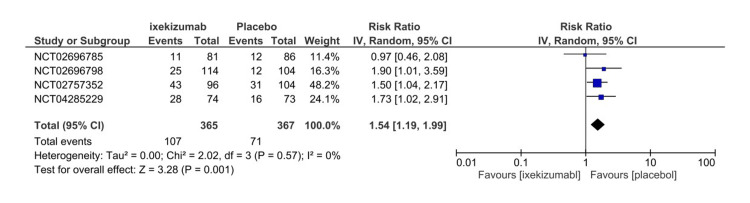
Nonserious adverse events forest plot CI, confidence interval; IV, inverse variance; RR, risk ratio [8-11]

**Figure 12:**
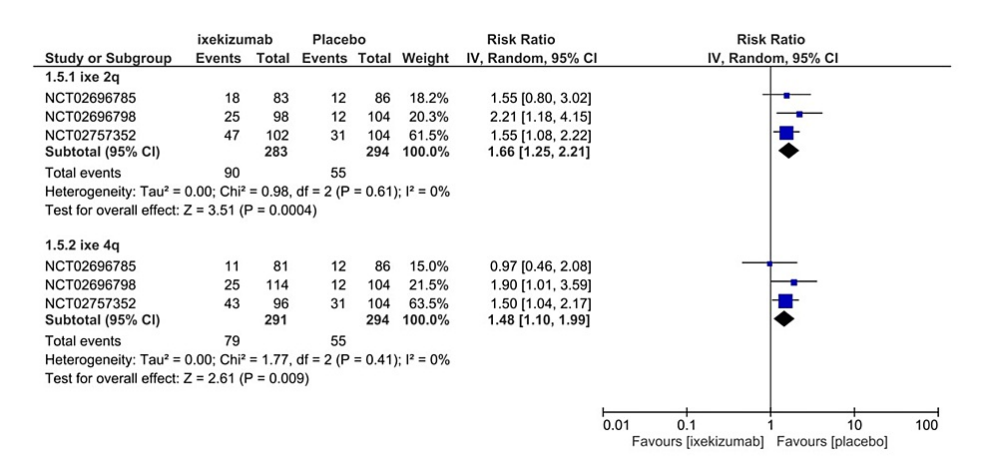
Nonserious adverse events subgroup forest plot CI, confidence interval; IV, inverse variance; RR, risk ratio; Q2W, every 2 weeks; Q4W, every 4 weeks [8-10]

All included studies assessed NSAEs from the start of the trial until the last administered dose or follow-up. NSAEs are defined as any event that does not result in death, major incapacitation, hospitalization, interfere significantly with participants’ normal life, or a congenital anomaly or birth defect in which no correlation of drug to the NSAEs must be established per the study protocol. The ixekizumab arm had significantly higher NSAEs than the placebo arm (107 versus 71) (RR = 1.54, 95% CI 1.19-1.99, P = 0.001, I2 = 0%) (Figure 11). Moreover, IXE80Q2W (RR = 1.66, 95% CI 1.25-2.21, P = 0.004, I2 = 0%) and IXE80Q4W (RR = 1.48, 95% CI 1.10-1.99, P = 0.001, I2 = 0%) arms demonstrated a significantly higher incidence of NSAEs (Figure 12). GRADE certainty of evidence was found to be rated as high for NSAEs (Table 3).

Mortality

Mortality was defined as any death that occurred during the trial regardless of the cause. None of the included studies reported any mortality.

Discussion

Our systematic review and meta-analysis assessed the efficacy and safety of ixekizumab for the treatment of axSpA. The ixekizumab arms demonstrated a significant improvement in the ASAS40 response in comparison to the placebo. This conclusion is consistent with data published in a previous meta-analysis, but our meta-analysis includes more recent RCTs [13]. Additionally, we assessed various endpoints including clinically significant endpoints (e.g., ASDAS) and radiographically significant endpoints (e.g., SPARCC score). Moreover, we assessed the certainty of the evidence through the GRADE criteria. A significant decrease in both the SPARCC score and ASDAS was noted in IXE80Q4W compared to the placebo, which reflects the improvement of axSpA. In contrast, IXE80Q4W reported a significant increase in the incidence of NSAEs.

Moreover, IXE80Q4W did not demonstrate a significant incidence of either SAEs or mortality, which is also consistent with the data published in the most recent meta-analysis [13]. IXE80Q4W in comparison to IXE80Q2W showed a higher incidence of SAEs, but it was insignificant.

The management of axSpA involves different treatment options ranging from NSAIDs, bDMARDs, and glucocorticoids (GCs) to physical therapy [14]. NSAIDs remain the rheumatologist’s first-line treatment for axSpA, as they have been shown to improve axSpA symptoms. However, continuous administration of NSAIDs has been linked to an increased risk of developing cardiovascular, gastrointestinal, and renal complications [6,14]. The latest generation of NSAIDs (cyclooxygenase 2) is more selective, which leads to a decrease in gastrointestinal side effects [6]. On the other hand, selective NSAIDs demonstrated a higher risk of developing hypertension, edema, and congestive heart failure [15]. bDMARDs, which include TNF-i therapy, are recommended for patients with a high disease activity index. TNF-i therapy is approved for the treatment of radiographic axial spondyloarthritis (axSpA) and for non-radiographic axSpA if there is evidence of inflammation on MRI or elevated CRP [16]. TNF-i safety was evaluated, and it demonstrated no significant risk of developing SAEs versus placebo [17]. Nevertheless, intolerance to the side effects or inadequate response to TNF-i necessitates the use of IL-17A inhibitors [16]. The efficacy of GCs for the management of axSpA was assessed in a recent systematic review in which it was difficult to evaluate due to the limited number of studies that included either systemic or local injections. Additionally, GCs have demonstrated some efficacy in systemic high-dose injections [18]. However, ASAS-EULAR recommends not administering GCs for prolonged periods [16].

Ixekizumab, an IL-17A inhibitor, has been proposed as a potential novel treatment for axSpA, but the insufficient number of high-quality RCTs remains a major hindrance to assessing both its safety and efficacy. Thus, more high-quality RCTs are required to establish the feasibility of ixekizumab for the management of axSpA.

We comprehensively analyzed ixekizumab in all four included studies for efficacy through the ASAS40, ASDAS, and SPRACC and investigated its safety by the incidence of SAEs, NSAEs, and mortality. We found that ixekizumab significantly improved the ASAS40, ASDAS, and SPRACC scores and improved the signs and symptoms of axSpA. Nonetheless, the finding of significant heterogeneity for ASDAS and SPRACC might impact the credibility of these results. However, two extension trials for ixekizumab for 52 weeks were concluded, and they demonstrated comparable results in comparison to 16-week trials [19]. Additionally, all included studies on ASDAS and SPARCC score favored the ixekizumab arm, in which the magnitude of mean change might be the source of this high heterogeneity. These findings of favorable outcomes for ixekizumab in comparison to placebo are consistent with published meta-analyses [13]. Thus, ixekizumab is a novel treatment for axSpA that shows promise as an efficacious drug for axSpA management protocols.

The most reported adverse events (AEs) were upper respiratory tract infection, injection site reaction, and nasopharyngitis. Ixekizumab incidence of NSAEs was significantly higher than the placebo group, but SAEs were insignificant in comparison to the placebo arm in which the risk for SAEs is similar to a previously published meta-analysis [13]. The mortality rate was unavailable due to the lack of events occurring during the RCTs. In theory, IL-17A inhibitors are immunomodulator medications that might cause SAEs, NSAEs, and mortality, so interpretation of their safety profile should be approached cautiously [19]. Additionally, IL-17A demonstrated an increased risk for AEs in moderate-to-severe plaque psoriasis [20].

As ixekizumab is a novel drug, more high-quality RCTs and, by extension, more elaborate systematic reviews should be conducted to further investigate its efficacy and safety. Reliable primary efficacy endpoints at different intervals and further diversity in terms of sex and ethnicity should be considered when conducting new RCTs. A funnel plot was unavailable in this study due to the number of included RCTs, which might hinder the visual interpretation of the funnel plot.

GRADE criteria were utilized to assess the quality of evidence. GRADE criteria are defined as the assessment of five major domains: risk of bias, imprecision, inconsistency, indirectness, and publication bias. The GRADE assessment would provide a greater degree of certainty to the evaluated evidence. Nevertheless, the clinical judgment of evidence is further required to reach an accurate assessment.

This study has some potential limitations. One limitation is the low number of RCTs conducted, which might be due to the novelty of the drug. Moreover, significant heterogeneity was noted for ASDAS and SPRACC scores, which could be due to methodological differences that led to high I2 hindering the interpretation of these results. High heterogeneity could be attributed to major differences in the magnitude of change in all included studies because all of them favored the intervention arm.

## Conclusions

To conclude, ixekizumab demonstrates an effective treatment option for axSpA patients who failed NSAID therapy. Moreover, it shows significant improvement in all primary endpoints such as ASAS40, ASDAS, and SPRACC scores. AEs of ixekizumab do exhibit conflicting evidence. On the one hand, ixekizumab SAEs are insignificant in comparison to placebo. Nevertheless, a significant incidence of NSAEs could hinder their safety. The frequency of ixekizumab administration does not significantly correlate with improved safety. However, IXE80Q2W shows an increase in ASAS40 events in comparison to IXE80Q4W. The GRADE criteria for quality assessment demonstrates a high certainty of evidence in most of the primary endpoints assessed. Further RCTs are required to explore different primary endpoints for axSpA management. Upcoming studies on ixekizumab should evaluate its efficacy and safety in comparison to current management options for axSpA.

## References

[1] McVeigh CM, Cairns AP (2006). Diagnosis and management of ankylosing spondylitis. BMJ.

[2] Stolwijk C, van Onna M, Boonen A, van Tubergen A (2016). Global prevalence of spondyloarthritis: a systematic review and meta-regression analysis. Arthritis Care Res (Hoboken).

[3] van der Heijde D, Baraliakos X, Gensler LS (2018). Efficacy and safety of filgotinib, a selective Janus kinase 1 inhibitor, in patients with active ankylosing spondylitis (TORTUGA): results from a randomised, placebo-controlled, phase 2 trial. Lancet.

[4] Machado P, Landewé R, Lie E, Kvien TK, Braun J, Baker D, van der Heijde D (2011). Ankylosing Spondylitis Disease Activity Score (ASDAS): defining cut-off values for disease activity states and improvement scores. Ann Rheum Dis.

[5] Panwar J, Tse SM, Lim L (2019). Spondyloarthritis research consortium of Canada scoring system for sacroiliitis in juvenile spondyloarthritis/enthesitis-related arthritis: a reliability, validity, and responsiveness study. J Rheumatol.

[6] Kroon F, Landewé R, Dougados M, van der Heijde D (2012). Continuous NSAID use reverts the effects of inflammation on radiographic progression in patients with ankylosing spondylitis. Ann Rheum Dis.

[7] Sieper J, Poddubnyy D (2017). Axial spondyloarthritis. Lancet.

[8] Deodhar A, van der Heijde D, Gensler LS (2020). Ixekizumab for patients with non-radiographic axial spondyloarthritis (COAST-X): a randomised, placebo-controlled trial. Lancet.

[9] Deodhar A, Poddubnyy D, Pacheco-Tena C (2019). Efficacy and safety of ixekizumab in the treatment of radiographic axial spondyloarthritis: sixteen-week results from a Phase III randomized, double-blind, placebo-controlled trial in patients with prior inadequate response to or intolerance of tumor necrosis factor inhibitors. Arthritis Rheumatol.

[10] van der Heijde D, Cheng-Chung Wei J, Dougados M (2018). Ixekizumab, an interleukin-17A antagonist in the treatment of ankylosing spondylitis or radiographic axial spondyloarthritis in patients previously untreated with biological disease-modifying anti-rheumatic drugs (COAST-V): 16 week results of a phase 3 randomised, double-blind, active-controlled and placebo-controlled trial. Lancet.

[11] (2020). A study of ixekizumab (LY2439821) in Chinese participants with radiographic axial spondyloarthritis. US). 2000 Feb 29: Identifier NCT04285229, S A Study of Ixekizumab (LY2439821) in Chinese Participants With Radiographic Axial Spondyloarthritis.

[12] van der Linden S, Valkenburg HA, Cats A (1984). Evaluation of diagnostic criteria for ankylosing spondylitis. A proposal for modification of the New York criteria. Arthritis Rheum.

[13] Wang P, Zhang S, Hu B, Liu W, Lv X, Chen S, Shao Z (2021). Efficacy and safety of interleukin-17A inhibitors in patients with ankylosing spondylitis: a systematic review and meta-analysis of randomized controlled trials. Clin Rheumatol.

[14] Moon KH, Kim YT (2014). Medical treatment of ankylosing spondylitis. Hip Pelvis.

[15] Sharma JN, Jawad NM (2005). Adverse effects of COX-2 inhibitors. ScientificWorldJournal.

[16] van der Heijde D, Ramiro S, Landewé R (2017). 2016 update of the ASAS-EULAR management recommendations for axial spondyloarthritis. Ann Rheum Dis.

[17] Hou LQ, Jiang GX, Chen YF (2018). The comparative safety of TNF inhibitors in ankylosing spondylitis—a meta-analysis update of 14 randomized controlled trials. Clin Rev Allergy Immunol.

[18] Dhir V, Mishra D, Samanta J (2021). Glucocorticoids in spondyloarthritis—systematic review and real-world analysis. Rheumatology (Oxford).

[19] Dougados M, Wei JC, Landewé R (2020). Efficacy and safety of ixekizumab through 52 weeks in two phase 3, randomised, controlled clinical trials in patients with active radiographic axial spondyloarthritis (COAST-V and COAST-W). Ann Rheum Dis.

[20] Erichsen CY, Jensen P, Kofoed K (2020). Biologic therapies targeting the interleukin (IL)-23/IL-17 immune axis for the treatment of moderate-to-severe plaque psoriasis: a systematic review and meta-analysis. J Eur Acad Dermatol Venereol.

